# B7-H4 reduction induced by *Toxoplasma gondii* infection results in dysfunction of decidual dendritic cells by regulating the JAK2/STAT3 pathway

**DOI:** 10.1186/s13071-022-05263-1

**Published:** 2022-05-03

**Authors:** Xinyue Sun, Hongbing Xie, Haixia Zhang, Zhidan Li, Houbao Qi, Chunyan Yang, Xianbing Liu, Liqin Ren, Yuzhu Jiang, Xuemei Hu

**Affiliations:** 1 Department of Immunology, Binzhou Medical University, Yantai, 264003 Shandong People’s Republic of China; 2 Department of Oral Biology, Binzhou Medical University, Yantai, 264003 Shandong People’s Republic of China; 3 Department of Medical Genetics and Cell Biology, Binzhou Medical University, Yantai, 264003 Shandong People’s Republic of China

**Keywords:** Maternal–fetal tolerance, Decidual DCs, Inhibitory molecule, B7-H4, *Toxoplasma gondii*, Abnormal pregnancy

## Abstract

**Background:**

Primary infection of *Toxoplasma gondii* can cause serious abnormal pregnancy outcomes such as miscarriage and stillbirth. Inhibitory molecule B7-H4 is abundantly expressed in dendritic cells (DCs) and plays an important role in maintaining immune tolerance. However, the role of B7-H4 in decidual DCs (dDCs) in *T. gondii*-induced abnormal pregnancy outcomes is not clear.

**Methods:**

We established *T. gondii-*infected abnormal pregnancy model in wild-type (WT) and B7-H4 knockout (B7-H4^−/−^) pregnant mice in vivo and cultured primary human dDCs in vitro. The abnormal pregnancy outcomes were observed and the expression of B7-H4, functional molecules (CD80, CD86, and MHC-II or HLA-DR), indoleamine 2,3-dioxygenase (IDO), cytokines (IL-10 and IL-12), and signaling molecules JAK2/STAT3 in dDCs was detected by flow cytometry and Western blot.

**Results:**

Our results showed that *T. gondii* infection significantly decreased B7-H4 expression in dDCs. In addition, B7-H4^−/−^ infected pregnant mice showed much more severe abnormal pregnancy outcomes than their counterparts. Importantly, B7-H4^−/−^ infection further regulated the expression of molecules (CD80, CD86, and MHC-II or HLA-DR), enzyme IDO, and cytokines (IL-10 and IL-12) in dDCs. We further discovered that B7-H4^−/−^ infection impairs the JAK2/STAT3 pathway, contributing to dDC dysfunction.

**Conclusions:**

Taken together, the results show that reduction of B7-H4 by *T. gondii* infection significantly modulates the decrease in cytokine IL-10 and enzyme IDO and the increase in cytokine IL-12, contributing to dDC dysfunction. Moreover, the JAK2/STAT3 pathway is involved in the regulation of B7-H4 by *T. gondii* infection and in the subsequent IDO and cytokine production, which ultimately contributes to abnormal pregnancy outcomes.

**Graphical Abstract:**

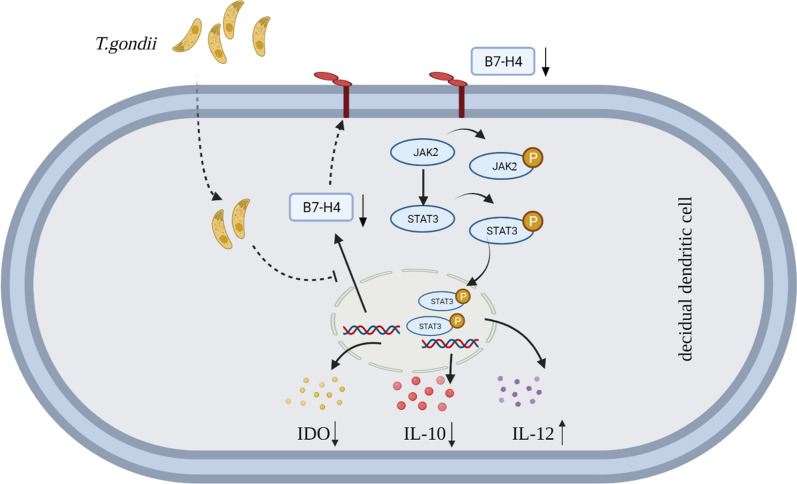

**Supplementary Information:**

The online version contains supplementary material available at 10.1186/s13071-022-05263-1.

## Background

*Toxoplasma gondii* is an obligate intracellular protozoan parasite that usually results in asymptomatic primary infection in healthy individuals [1]. However, immunocompromised individuals are susceptible to developing severe disease [2]. Pregnant women in their first trimester with a primary infection of *T. gondii* are particularly susceptible because the parasite can be vertically transmitted, resulting in a number of serious adverse pregnancy outcomes, including miscarriage, stillbirth, fetal growth restriction, and congenital toxoplasmosis [3]. Our previous studies revealed that the dysfunction of several decidual immune cells by *T. gondii* infection at the maternal–fetal interface contributed to adverse pregnancy outcomes in women as well as in a mouse model [4–6]. However, the detailed molecular mechanism of abnormal pregnancy during *T. gondii* infection still requires further clarification.

The immune microenvironment of the maternal–fetal interface plays an important role in sustaining a normal pregnancy and is composed of several different immune cells, functional molecules, and cytokines [7]. Decidual immune cells include dendritic cells (DCs), natural killer (NK) cells, macrophages, and regulatory T cells (Tregs) [8, 9]. Decidual DCs (dDCs) synthesize and secrete several immunosuppressive factors including interleukin 10 (IL-10) and indoleamine 2,3-dioxygenase (IDO), which contribute to sustaining maternal–fetal tolerance [8, 9], thus serving a vital function in normal pregnancy [10, 11].

Mammalian DCs are divided into at least two subsets, described as myeloid DCs (mDCs) and plasmacytoid DCs (pDCs) [12]. During normal early pregnancy in humans, DCs of the decidua include blood dendritic cell antigen (BDCA)1^+^CD19^−^CD14^−^ myeloid DC type 1 (MDC1), BDCA3^+^CD14^−^ myeloid DC type 2 (MDC2), and BDCA2^+^CD123^+^ (PDC), which all exert a tolerance function [13]. Murine dDCs are also generally divided into two distinct subsets: mDCs (CD11c^+^CD8a^−^) and lymphoid DCs (CD11c^+^CD8a^+^) [14]. Murine mDCs are identified as immature DCs characterized by the low expression of MHC-II, CD80, CD86, and pro-inflammatory cytokine IL-12, with high expression of anti-inflammatory cytokine IL-10 and intracellular enzyme IDO, which contribute to sustaining maternal–fetal immune tolerance [8, 15]. During pregnancy, murine mDCs are the main dDC subset at the maternal–fetal interface, and these contribute to tolerance that is crucial in maintaining a normal pregnancy [16]. Furthermore, pDCs also contribute to the maintenance of pregnancy at the maternal–fetal interface by producing lower IL-12 in the decidua than pDCs in the peripheral blood [8]. Studies have shown that several immunosuppressive molecules (B7-H4, B7-H3, LILRB4) expressed on mDCs participate in maternal–fetal tolerance [8, 17, 18].

B7-H4, also known as B7x, B7S1, or VTCN1, is a co-inhibitory molecule mainly expressed on professional antigen-presenting cells (APCs), and it is involved in the negative regulation of T-cell-mediated immune responses [19–21]. B7-H4 is widely expressed in various normal tissues, including the placenta and liver [22], as well as mDCs and pDCs during the first trimester of normal pregnancy [23]. Our previous studies confirmed that *T. gondii* infection reduces the expression of LILRB4 on dDCs, resulting in dDC dysfunction in contributing to maternal–fetal tolerance [24]. Whether *T. gondii* infection can affect the level of B7-H4 expression on dDCs, and whether this leads to further dysregulation of dDCs, remains unclear. We therefore used primary human dDCs and B7-H4^−/−^ pregnant female mice to explore the effect of *T. gondii* infection on B7-H4 expression in dDCs and to elucidate immune molecular mechanisms of dDC dysfunction.

## Methods

### Animal models

C57BL/6 wild-type (WT) mice (8–10 weeks old) were purchased from Pengyue Laboratory Animal Technology Co., Ltd. (Jinan, China). B7-H4-deficient (B7-H4^−/−^) C57BL/6 mice were purchased from Bioray Laboratories Inc. (Nanjing, China). All mice were kept under specific-pathogen-free (SPF) conditions at 22–26 °C, 50–60% relative humidity, and a 12 h light/dark cycle with sufficient aseptic water and food. Following overnight cohabitation of males with females, the visualization of a plug was designated as gestational day (Gd) 0 of pregnancy.

### The genotype of B7-H4^−/−^ mice

Genomic DNA was extracted from the tails of mice using a tissue DNA extraction kit (GeneRay, China). Polymerase chain reaction (PCR) was used to synthesize complementary DNA (cDNA). After initial denaturation (3 min at 95 °C), PCR was performed with 40 amplification cycles of denaturation for 30 s at 95 °C, annealing for 30 s at 55 °C, and extension for 60 s at 72 °C, followed by a final extension for 5 min at 72 °C. PCR products were separated by electrophoresis in 2% agarose gels, and sizes were estimated using Trans DNA Marker I (100–700 base pairs (bp); TransGen Biotech, China). Gels were stained with GelStain (10,000×; TransGen Biotech, China) to visualize DNA. Primers for PCR amplification were as follows: forward: GGCAAAGACGACCTCTCACA; reverse: CCCTTTGCCTTTTGAGGTGC. The expected PCR product sizes were 160 bp (heterozygote), 400 and 160 bp (mutant), and 400 bp (wild-type). Homozygous B7-H4^−/−^ mice were continually bred for the duration of the study.

### Preparation of *T. gondii* RH tachyzoites

*Toxoplasma gondii* tachyzoites were maintained in HEp-2 cells in minimum essential medium (MEM) (HyClone Laboratories, Logan, UT, USA), 5% fetal bovine serum (FBS; Gibco, Waltham, MA, USA), and 100 IU/ml penicillin/streptomycin (Sigma-Aldrich, St. Louis, MO, USA). After culture, HEp-2 cells were centrifuged at 1500 rpm (433×*g*) for 10 min, and then the supernatant was centrifuged at 4000 rpm (3082×*g*) for 7 min to purify tachyzoites. Purified tachyzoites were counted using a Neubauer chamber, resuspended, and cultured in MEM. Experiments were performed in biosafety level 2 (BSL-2) laboratories. All consumable labware and liquids contaminated with tachyzoites were sterilized and autoclaved immediately.

### Infection and pregnancy outcomes

WT pregnant female mice were randomly divided into two groups. One group (infected group) was inoculated intraperitoneally (i.p.) with 400 tachyzoites in 200 μl sterile phosphate-buffered saline (PBS) on Gd 8. The other group (uninfected group) was inoculated with 200 μl PBS as a control. In addition, B7-H4^−/−^ pregnant female mice were inoculated i.p. with the same number of tachyzoites as the WT infected group. Mice were euthanized 6 days after *T. gondii* infection (Gd14) and pregnancy outcomes were observed. Uteri of pregnant female mice were removed, and fetuses were extracted. The numbers of both normal and abnormal fetuses were counted. Abnormal fetuses were characterized by small size, necrotic and hemorrhagic appearance, or complete resorption compared with normal fetuses. The adverse pregnancy rate was calculated as the ratio of abnormal fetus numbers to total fetus numbers. Weights of placentae and fetuses were determined by electronic balance and averaged.

### Pathology assessments

Placentae and uteri from each of the three groups were collected after euthanasia on Gd 14; uteri were washed three times in PBS and immediately fixed with 4% paraformaldehyde. After fixation, tissues were washed in tap water and dehydrated in a graded ethanol series of 30%, 50%, and 70%, followed by paraffin embedding with standard methods. Sections were made and stained with hematoxylin and eosin (H&E; Shanghai Novland Co., Ltd., China) according to the manufacturer’s instructions. The images were obtained at 20× magnification.

### Scanning electron microscopy (SEM)

On Gd 14, pregnant female mice from all three groups were euthanized, and uteri were harvested. The fetuses were removed, washed five times in 0.1 M phosphate buffer, and fixed in 2.5% phosphate glutaraldehyde buffer at 4 °C for two days. Then, the fetuses were dehydrated with a gradient ethanol series every 15 min. Samples were dried by the critical point technique (Quorum K850), attached to the specimen scaffold, and coated with gold particles by ion sputter coating (Quorum Q150RS). All specimens were observed using a scanning electron microscope (Zeiss EVO LS15) operated at 10 kV, and images were obtained by SmartSEM user interface software.

### Cell preparation of mice

Uteri and placentae were carefully separated from pregnant female mice after euthanasia on Gd 14 and were washed twice in ice-cold PBS. Tissues were then cut into 1–3 mm pieces, and single-cell suspensions were obtained by filtration through 48 µm sterile nets. Mononuclear cells were obtained using Ficoll density gradient centrifugation in mouse lymphocyte separation medium (TBD Science, China) and used for flow cytometry analysis.

### Collection of human clinical samples

Clinical samples of decidual tissues were collected in the Department of Obstetrics and Gynecology of Yantai Affiliated Hospital of Binzhou Medical University and the Yantai Zhifu District Maternal and Child Health Hospital. Tissues were washed five to eight times with PBS, and the villi were removed from the tissues under sterile conditions. Decidual tissues were delivered to our laboratory within 2 h and were kept in Dulbecco's modified Eagle medium (DMEM)/high-glucose medium (HyClone, USA) with 100 IU/ml penicillin and 100 μg/ml streptomycin (Sigma-Aldrich, USA).

### Purification of human dDCs

Decidual tissue was isolated and cleaned by shaking in ice-cold PBS four times before tissue was cut into 0.1 cm pieces. Single-cell suspensions were isolated by incubating the tissues in the digestion buffer (RPMI, 1 mg/ml Collagenase IV and DNase I) for 60 min, then filtrated through 48 µm sterile nets. Mononuclear cells were isolated by density gradient centrifugation using a human lymphocyte separation medium (TBD Science) at 2000 rpm for 20 min at 20 °C. Purification of dDCs from monocytes was performed using the EasySep Human Myeloid DC Enrichment Kit (STEMCELL Technologies, Canada) according to the manufacturer’s instructions, with > 95% purity ensured for experiments. The marker of purified dDCs was negative selection of lineage (CD3, CD14, CD19, CD20, CD34, CD56). The purified dDCs were used for Western blot analysis.

### Human dDC culture and treatment

To test the effects of B7-H4 expression on human dDCs during *T. gondii* infection, approximately 1.5 × 10^6^ purified human dDCs were divided equally into three groups: uninfected, infected, and B7-H4-neutralized infected groups. In the B7-H4-neutralized infected group, dDCs were preincubated with anti-B7-H4 monoclonal antibody (mAb; 10 μg/ml) (eBioscience, San Diego, CA, USA) for 2 h. *Toxoplasma gondii* tachyzoites were added to the infected group and the B7-H4-neutralized infected group at a ratio of 2:1 (*T. gondii*: cells) followed by incubation for 24 h.

To test the role of STAT3 on enzyme and cytokine expression in dDCs during *T. gondii* infection, purified human dDCs were equally divided into four groups: uninfected, infected, STAT3-inhibitor, and STAT3-inhibitor infected groups. Cells of the STAT3-inhibitor and the STAT3-inhibitor infected groups were preincubated with Stattic (HY-13818, MedChem Express), a STAT3 inhibitor, which inhibited STAT3 phosphorylation. After 2 h, *T. gondii* tachyzoites were added to the STAT3-inhibitor infected group and the infected group at the same time as described above.

### Flow cytometry

Mouse decidual mononuclear cells or human DCs were incubated with corresponding mAbs at 4 °C in the dark for 30 min and then washed once. Cells were first incubated with antibodies against the cell surface proteins B7-H4, CD80, CD86, and MHC-II (mouse) or HLA-DR (human), fixed using a fixation and permeabilization kit (eBioscience, USA) according to the manufacturer’s instructions, and incubated with antibodies of intracellular proteins (IDO, IL-10, or IL-12). To analyze cytokines, cells were cultured for 4–6 h in a leukocyte activation cocktail (eBioscience, USA) before adding mAbs of cytokines. Analysis was performed using a FACSCanto II instrument (Becton Dickinson [BD], Franklin Lakes, NJ, USA) and FlowJo v.10.3 software (FlowJo, LLC, Ashland, OR, USA).

The following mouse-specific mAbs were used for flow cytometry: FITC-conjugated anti-CD11c, APC-conjugated anti-IDO (all from eBioscience, USA), PerCP-Cy5.5-conjugated anti-CD8α, PE-conjugated anti-CD80, PE-conjugated anti-CD86, PE-conjugated anti-MHC-II, PE-conjugated anti-IL-10, and PE-conjugated anti-IL-12 (all from BD, USA), and APC-conjugated anti-B7-H4 (BioLegend, San Diego, CA, USA).

The following human-specific mAbs were used: BV421-conjugated anti-BDCA1, BV421-conjugated anti-BDCA2, and BV421-conjugated anti-BDCA3, PE-Cy7-conjugated anti-CD14, PE-Cy7-conjugated anti-CD19, PE-Cy7-conjugated anti-CD123 (all from BD, USA), APC-conjugated anti-B7-H4 (BioLegend, USA), PE-conjugated anti-CD80, PE-conjugated anti-CD86, and PE-conjugated anti-HLA-DR (all from eBioscience, USA).

### Immunofluorescence (IF) imaging

Purified human dDCs from uninfected, infected, or B7-H4-neutralized infected groups were fixed in 4% paraformaldehyde for 30 min and blocked with goat serum for 1 h at 37 °C. dDCs were incubated overnight at 4 °C with a primary antibody. After washing three times with PBS, cells were incubated with secondary antibodies for 1 h at 37 °C, stained with 4′,6-diamidino-2-phenylindole (DAPI) for 15 min, and again washed three times with PBS. Finally, cells were examined using confocal microscopy (Zeiss LSM 880).

### Western blot analysis

A total of 1 × 10^7^ cells of each group were lysed with pre-cooled RIPA lysis buffer with the serine protease inhibitor phenylmethylsulfonyl fluoride (PMSF) and protein phosphatase inhibitor. After cooling on ice for 30 min, lysates were centrifuged for 20 min at 12,000×*g*. The supernatant was harvested and analyzed for protein concentration using a BCA [bicinchoninic acid] protein assay kit (Beyotime). Protein samples were then boiled in 5× loading buffer (Beyotime) for 8 min. Samples containing 30 μg of total protein were resolved by 10% sodium dodecyl sulfate–polyacrylamide gel electrophoresis (SDS-PAGE), and proteins were transferred to a polyvinylidene fluoride (PVDF) membrane (Millipore Corporation, Billerica, MA, USA). Each membrane was blocked with 5% nonfat milk in 1× Tris-buffered saline with Tween (TBST) for 2–3 h at room temperature. Each membrane was then incubated with a primary antibody overnight at 4 °C and a secondary antibody for 2 h at room temperature. The membrane was washed six times with 1× TBST for 30 min. Then, membranes were quantified with an enhanced chemiluminescence (ECL) detection kit (Roche, Ltd., Switzerland). Protein levels were analyzed by ImageJ software (Rawak Software, Inc., Germany).

### Statistical analysis

All data are presented as means ± standard deviations (SD). The statistical analyses were conducted using the GraphPad Prism 8 statistical software package. Differences in means were determined and analyzed by unpaired *t*-tests. (*/^△^*P* < 0.05; **/^△△^*P* < 0.01; ***/^△△△^*P* < 0.001 or ****/^△△△^*P* < 0.0001), *P* < 0.05 were considered statistically significant.

## Results

### B7-H4 expression on dDC subsets decreased after *T. gondii* infection

**Fig. 1:**
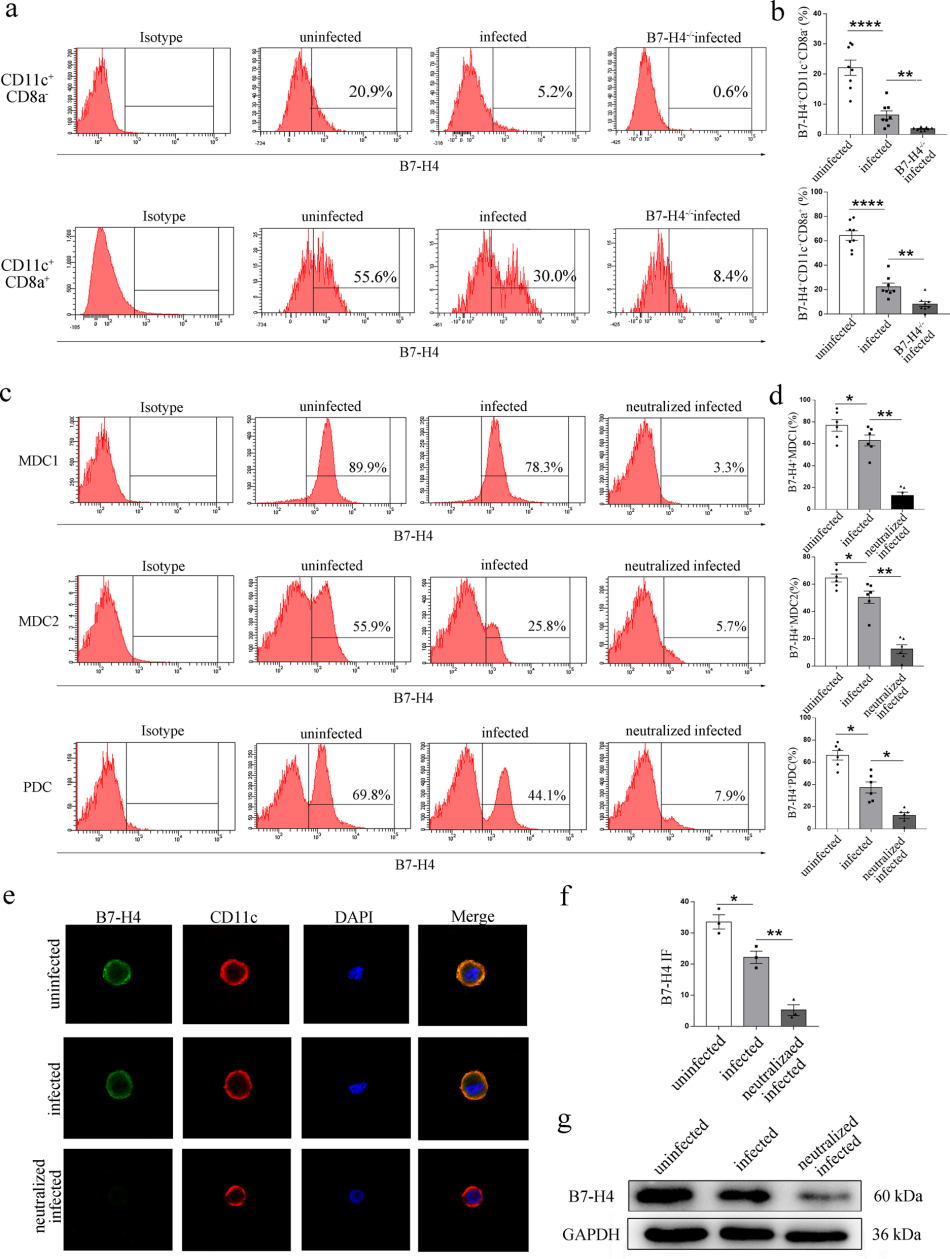
Expression of B7-H4 on human and mice dDC subsets infected with *T. gondii*. **a** Flow cytometry analyses of B7-H4 levels in mouse CD11c^+^CD8a^−^ and CD11c^+^CD8a^+^ dDCs from WT uninfected, WT infected, and B7-H4^−/−^ pregnant female mice. **b** Histogram analysis of B7-H4 expression changes in CD11c^+^CD8a^−^ and CD11c^+^CD8a^+^ dDCs from WT uninfected, WT infected, or B7-H4^−/−^ infected groups of mice. **c** Flow cytometry analyses of B7-H4 levels on human decidual MDC1, MDC2, and PDC subsets from uninfected, infected, and B7-H4-neutralized infected groups. **d** Histogram analysis of B7-H4 expression changes in human decidual MDC1, MDC2, and PDC subsets from uninfected, infected, and B7-H4-neutralized and infected groups. **e** Representative images of IF of B7-H4 (green) expression levels in purified human dDCs from uninfected, infected, or B7-H4-neutralized infected groups. DAPI (blue) labeling defined the morphology of nuclei. Scale bars, 10 μm. **f** Histogram analysis of B7-H4 expression changes on purified human dDCs by immunofluorescence intensity among three groups. **g** Western blot analysis of B7-H4 levels in purified human dDCs from uninfected, infected, and B7-H4-neutralized infected groups. IFI, immunofluorescence intensity. Data shown are means ± SD. At least six samples of human decidua in each group were assayed individually in flow cytometry and at least three samples of human decidua in each group were assayed individually in Western blot and IF by paired *t*-test for uninfected vs infected group, infected vs B7-H4-neutralized group. At least eight pregnant female mice in each group were assayed individually by unpaired *t*-test for uninfected vs infected group, infected vs B7-H4^−/−^infected group

Firstly, we used flow cytometry to analyze the effect of *T. gondii* infection on the expression of B7-H4 in different mouse dDC subsets. In addition, IF and Western blot were used to examine the expression of B7-H4 in human dDCs. The results showed that B7-H4 expression on dDCs was significantly reduced (CD11c^+^CD8a-: *t*-test, *t*_(14)_ = 5.444, *P* < 0.0001; CD11c^+^CD8a^+^: *t*-test, *t*_(14)_ = 8.553, *P* < 0.0001; MDC1: *t*-test, *t*_(5)_ = 3.981, *P* = 0.0105; MDC2: *t*-test, *t*_(5)_ = 3.929, *P* = 0.0111; MDC2: *t*-test, *t*_(5)_ = 3.284, *P* = 0.0219; IF: *t*-test, *t*_(2)_ = 6.035, *P* = 0.0264) after *T. gondii* infection in vivo (Fig. 1a, b; Additional file 1: Fig. S1a) and in vitro (Fig. 1c–g; Additional file 1: Fig. S1b).

### B7-H4^−/−^ pregnant female mice have more severe abnormal pregnancy outcomes after *T. gondii* infection than those of WT pregnant female mice

**Fig. 2:**
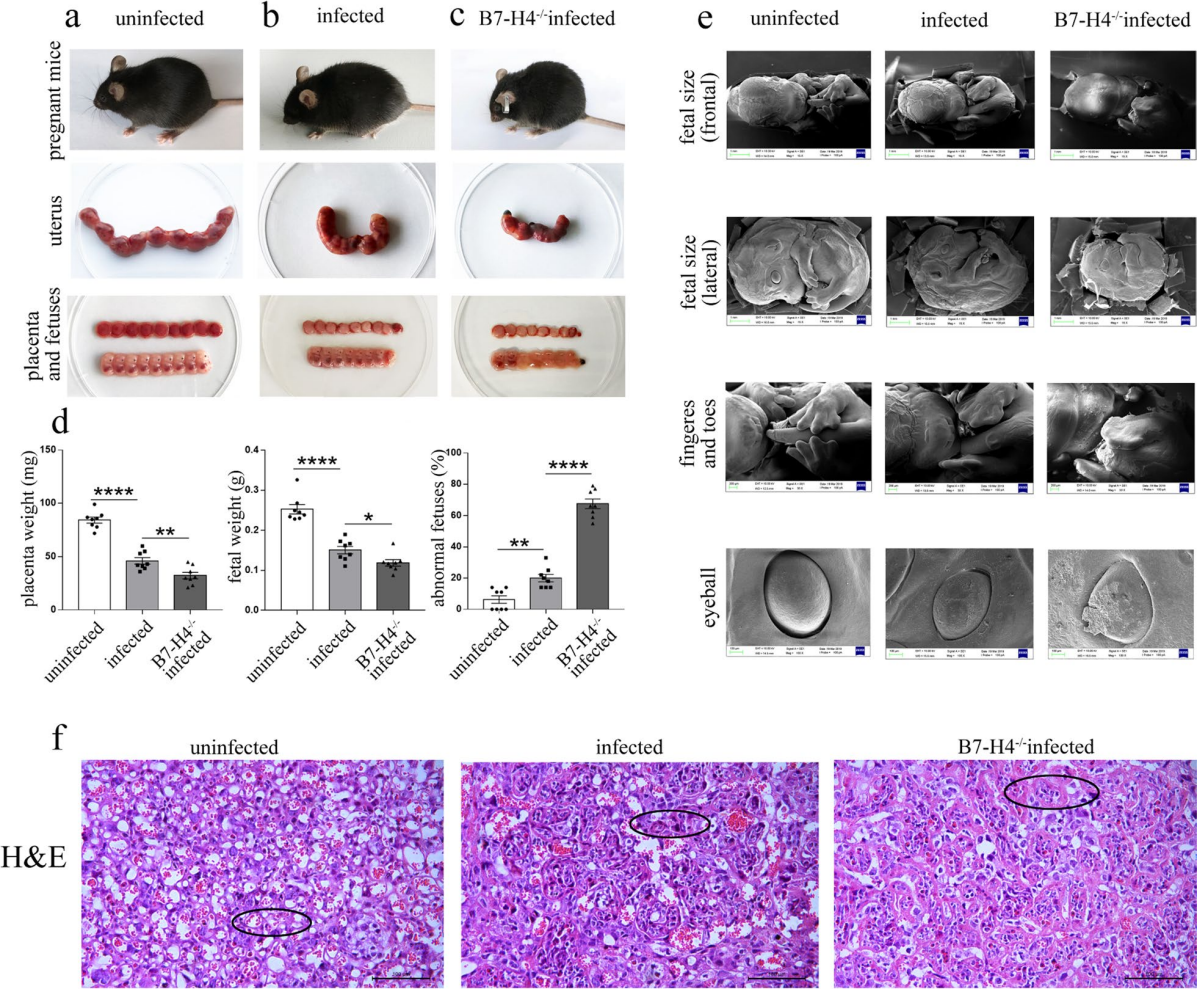
Effect of B7-H4 on abnormal pregnancy outcomes caused by *T. gondii* infection in pregnant female mice. **a** WT uninfected pregnant female mice had good mental states, bright hair, and well-developed placentas and fetuses. **b** WT infected pregnant female mice had poor mental conditions, dull hair, and small placentas and fetuses with poor blood supply. **c** B7-H4^−/−^ infected pregnant female mice were in a poor mental state with dull hair and dim eyes, and had more stillbirths and absorptive fetuses. **d** The weight of the placenta and fetus and the resorption rate in WT uninfected, WT infected, and B7-H4^−/−^ infected pregnant female mice. **e** Representative scanning electron microscopy (SEM) images of the fetal development showing fetal size, eyeballs, fingers, and toes. **f** Representative hematoxylin and eosin (H&E) staining images of placentas from WT uninfected, WT infected, and B7-H4^−/−^ infected pregnant female mice, showing fibrinoid necrosis of blood vessels (black circle). Data shown are means ± SD. At least eight pregnant female mice in each group were assayed individually by unpaired *t*-test for uninfected vs infected group, infected vs B7-H4^−/−^infected group

To explore the role of B7-H4 in abnormal pregnancy caused by *T. gondii* infection, *T. gondii-*infection and B7-H4^−/−^ pregnant female mice were established. WT and B7-H4^−/−^ pregnant female mice were inoculated intraperitoneally (i.p.) with 400 tachyzoites in 200 μl PBS on Gd 8. The results revealed that WT pregnant female mice with *T. gondii* infection showed abnormal pregnancy outcomes, with poor mental conditions, dull hair, small placentae and fetuses, partially abnormal fetuses, and poor blood supply. Placentae (*t*-test: *t*_(14)_ = 9.16, *P* < 0.0001) and fetuses (*t*-test: *t*_(14)_ = 6.919, *P* < 0.0001) were significantly reduced, and abnormal pregnancy rates were significantly increased (*t*-test: *t*_(14)_ = 4.095, *P* = 0.0011) (Fig. 2a–d). Moreover, B7-H4^−/−^ infected pregnant female mice showed more serious outcomes, and the placental (*t*-test: *t*_(14)_ = 3.21, *P* = 0.0063) and fetal weights (*t*-test: *t*_(14)_ = 2.568, *P* = 0.0223) were significantly decreased compared to those of the WT infected group (Fig. 2a–d). Further, in B7-H4^−/−^ infected pregnant female mice, stillbirths, abnormal fetuses, and abnormal pregnancy rates were significantly increased (*t*-test: *t*_(14)_ = 12.2, *P* < 0.0001) relative to WT infected pregnant female mice (Fig. 2a–d). Scanning electron microscopy (SEM) revealed that fetuses from B7-H4^−/−^ infected pregnant female mice were more hypogenetic than those from WT infected pregnant female mice (Fig. 2e); in addition, B7-H4^−/−^ infected pregnant female mice showed smaller body size, unformed fingers and toes, and abnormal eyeball development. H&E-stained placental sections revealed that placental blood vessels in WT uninfected pregnant female mice were well developed, and the vascular lumen was open and reticulated with abundant red blood cells. Placental blood vessels of WT infected pregnant female mice showed fibrinoid degeneration, thickened walls, narrowed lumina, and decreased red blood cells. In addition to having these abnormalities, placental blood vessels in B7-H4^−/−^ infected pregnant female mice developed fibrinoid necrosis (Fig. 2f). These observations suggest that the reduction of B7-H4 expression plays an important role in adverse pregnancy outcomes caused by *T. gondii* infection.

### Reduction of B7-H4 after *T. gondii* infection was associated with the changes in functional molecules on dDCs

**Fig. 3:**
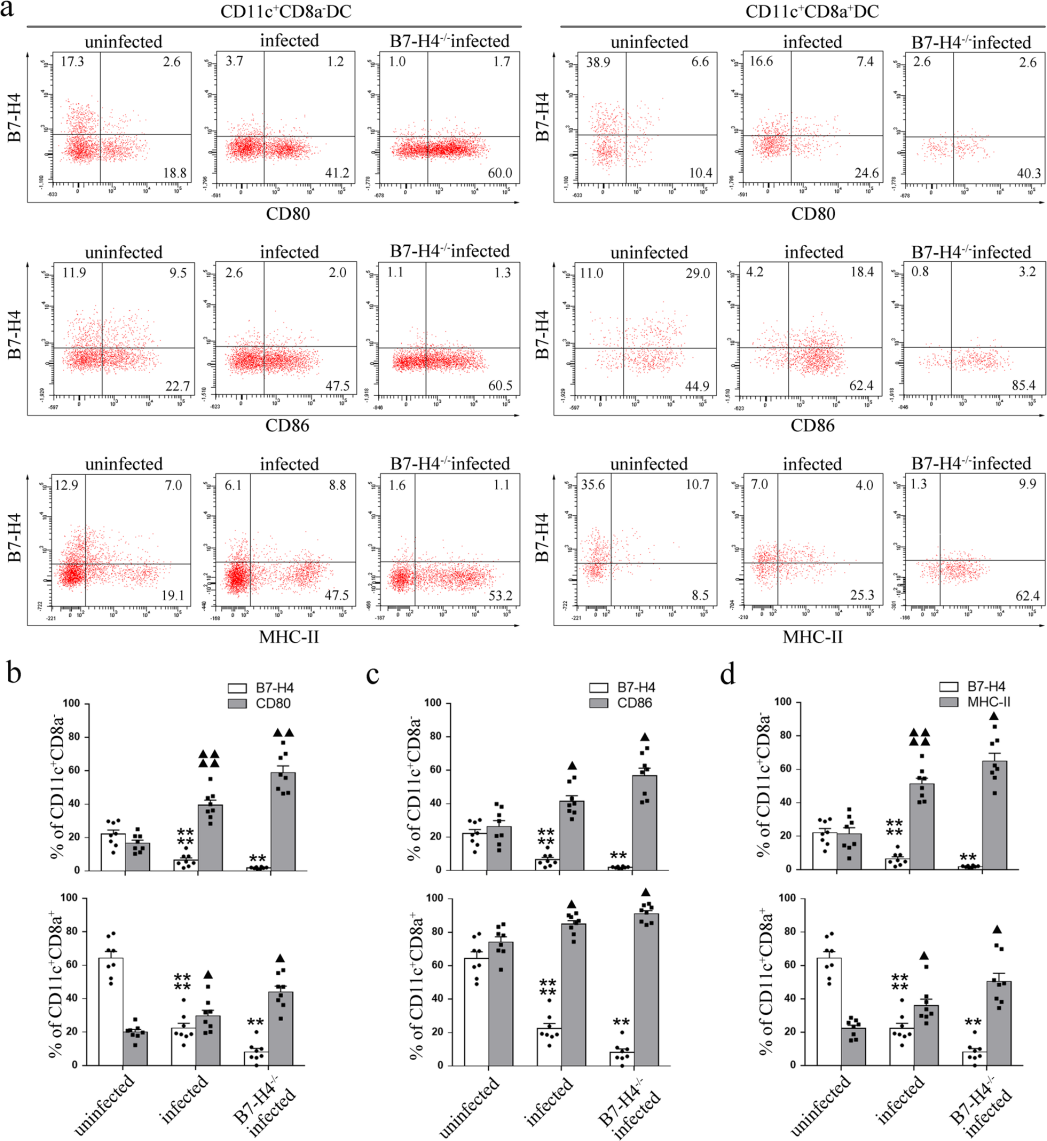
Reduction of B7-H4 on mouse dDCs cells by *T. gondii* infection related to the expression of MHC-II and co-stimulatory molecules CD80 and CD86. **a** Flow cytometry analyses of CD80, CD86, MHC-II, and B7-H4 levels in CD11c^+^CD8a^−^ and CD11c^+^CD8a^+^ dDCs from WT uninfected, WT infected, and B7-H4^−/−^ infected groups of pregnant female mice. **b**–**d** Histogram analysis of CD80, CD86, MHC-II, and B7-H4 expression changes in CD11c^+^CD8a^−^ and CD11c^+^CD8a^+^ dDCs from WT uninfected, WT infected, and B7-H4^−/−^ infected pregnant female mice. Data shown are means ± SD. At least eight pregnant female mice per group were assayed individually by unpaired *t*-test; uninfected vs infected group, infected vs B7-H4^−/−^ infected group

**Fig. 4:**
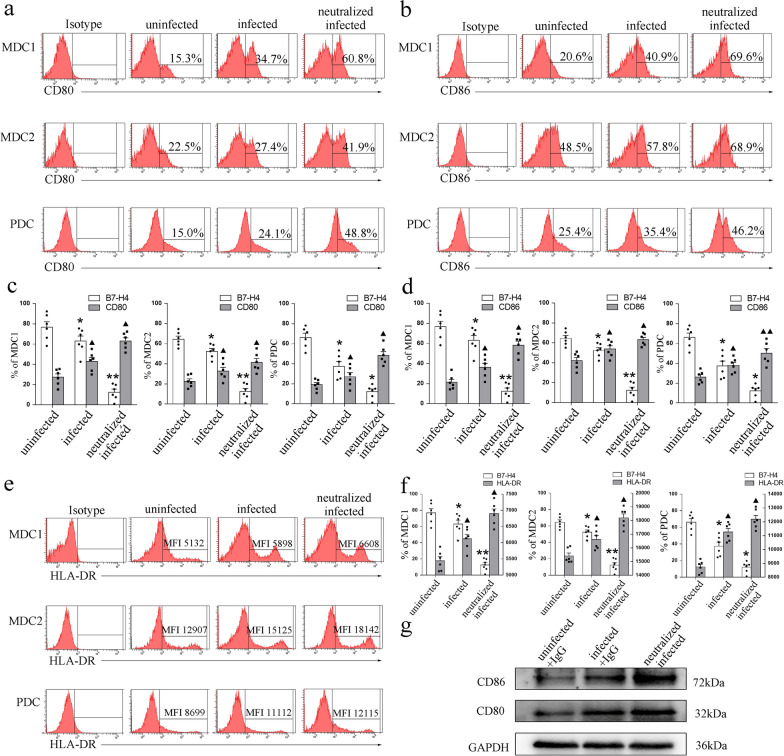
Reduction of B7-H4 on human dDCs by *T. gondii* infection associated with the expression of HLA-DR and co-stimulatory molecules CD80 and CD86. **a**, **b**, **e** Flow cytometry analyses of CD80, CD86, and HLA-DR levels on human decidual MDC1, MDC2, and PDC subsets from uninfected, infected, and B7-H4-neutralized infected groups. MFI = mean fluorescence intensity. **c**, **d**, **f** Histogram analysis of CD80, CD86, HLA-DR, and B7-H4 expression changes in human decidual MDC1, MDC2, and PDC subsets from uninfected, infected, and B7-H4-neutralized infected groups. **g** Western blot analysis of CD80 and CD86 levels in purified human dDCs from uninfected, infected, and B7-H4-neutralized infected groups. All data shown are means ± SD. At least six samples of human decidua in each group were assayed individually in flow cytometry and at least three samples of human decidua in each group were assayed individually in Western blot by paired *t*-test for uninfected vs infected group, infected vs B7-H4-neutralized group

To investigate whether the decrease in B7-H4 caused by *T. gondii* infection affects the expression of functional surface molecules on dDCs, the expression level of CD80, CD86, and MHC-II (mice) or HLA-DR (humans) on dDCs was analyzed by flow cytometry and Western blot. B7-H4-neutralizing antibody was added to *T. gondii*-infected human dDCs to block the functional pathway for investigating the effect of B7-H4 on functional molecules of dDCs during *T. gondii*-infected abnormal pregnancy outcomes. Flow cytometry showed that in *T. gondii*-infected and WT pregnant mice, the levels of membrane-functional molecules CD80 (CD11c^+^CD8a^−^: *t*-test: *t*_(14)_ = 6.562, *P* < 0.0001, CD11c^+^CD8a^+^: *t*-test: *t*_(14)_ = 2.652, *P* = 0.0189); CD86 (CD11c^+^CD8a^−^: *t*-test: *t*_(14)_ = 2.959, *P* = 0.0104, CD11c^+^CD8a^+^: *t*-test: *t*_(14)_ = 2.83, *P* = 0.0134) and MHC-II (CD11c^+^CD8a-: *t*-test: *t*_(14)_ = 6, *P* < 0.0001, CD11c^+^CD8a^+^: *t*-test: *t*_(14)_ = 2.955, *P* = 0.0104) were significantly increased, but B7-H4 levels were decreased in both CD11^+^CD8a^−^ (*t*-test: *t*_(14)_ = 5.444, *P* < 0.0001) and CD11c^+^CD8a^+^ (*t-*test: *t*_(14)_ = 8.553,* P* < 0.0001) subsets of dDCs compared with those of uninfected WT pregnant female mice (Fig. 3a–d). Moreover, higher levels of membrane-functional molecules CD80 (CD11c^+^CD8a-: *t-*test: *t*_(14)_ = 3.825, *P* = 0.0019, CD11c^+^CD8a^+^:*t*-test: *t*_(14)_ = 2.976, *P* = 0.0100); CD86 (CD11c^+^CD8a-: *t*-test: *t*_(14)_ = 2.808, *P* = 0.0140, CD11c^+^CD8a^+^: *t*-test: *t*_(14)_ = 2.289, *P* = 0.0382); MHC-II (CD11c^+^CD8a-: *t*-test: *t*_(14)_ = 2.36, *P* = 0.0333, CD11c^+^CD8a^+^: *t*-test: *t*_(14)_ = 2.334, *P* = 0.0350) were expressed on the dDCs of B7-H4^−/−^infected pregnant female mice (Fig. 3a–d) than on those of WT infected pregnant female mice. Similarly, CD80 (MDC1: *t*-test: *t*_(5)_ = 3.02, *P* = 0.0294, MDC2: *t*-test: *t*_(5)_ = 2.6, *P* = 0.0483, PDC: *t*-test: *t*_(5)_ = 3.685, *P* = 0.0142), CD86 (MDC1: *t*-test: *t*_(5)_ = 3.239, *P* = 0.0230, MDC2: *t*-test: *t*_(5)_ = 3.253, *P* = 0.0226, PDC: *t*-test: *t*_(5)_ = 2.738, *P* = 0.0409), and HLA-DR(MDC1: *t*-test: *t*_(5)_ = 2.578, *P* = 0.0496, MDC2: *t*-test: *t*_(5)_ = 3.439, *P* = 0.0185, PDC: *t*-test: *t*_(5)_ = 3.080, *P* = 0.0275) expression of human dDCs was significantly increased after *T. gondii* infection (Fig. 4a–f) and CD80 (MDC1: *t*-test: *t*_(5)_ = 3.151, *P* = 0.0253, MDC2: *t*-test: *t*_(5)_ = 2.844, *P* = 0.0361, PDC: *t*-test: *t*_(5)_ = 2.789, *P* = 0.0385), CD86 (MDC1: *t*-test: *t*_(5)_ = 3.769, *P* = 0.0130, MDC2: *t*-test: *t*_(5)_ = 2.622, *P* = 0.0470, PDC: *t*-test: *t*_(5)_ = 5.656, *P* = 0.0024) and HLA-DR (MDC1: *t*-test: *t*_(5)_ = 2.915, *P* = 0.0332, MDC2: *t*-test: *t*_(5)_ = 2.679, *P* = 0.439, PDC: *t*-test: *t*_(5)_ = 2.826, *P* = 0.0368) further upregulated in the B7-H4-neutralized infected group (Fig. 4a–f). As shown in Fig. 4g, Western blot also demonstrated that the expression of CD80 and CD86 in human dDCs was increased after *T. gondii* infection, and their levels were further increased in the B7-H4-neutralized infected group.

### Reduction of B7-H4 on dDCs by *T. gondii* infection was associated with the expression of the intracellular IDO

**Fig. 5:**
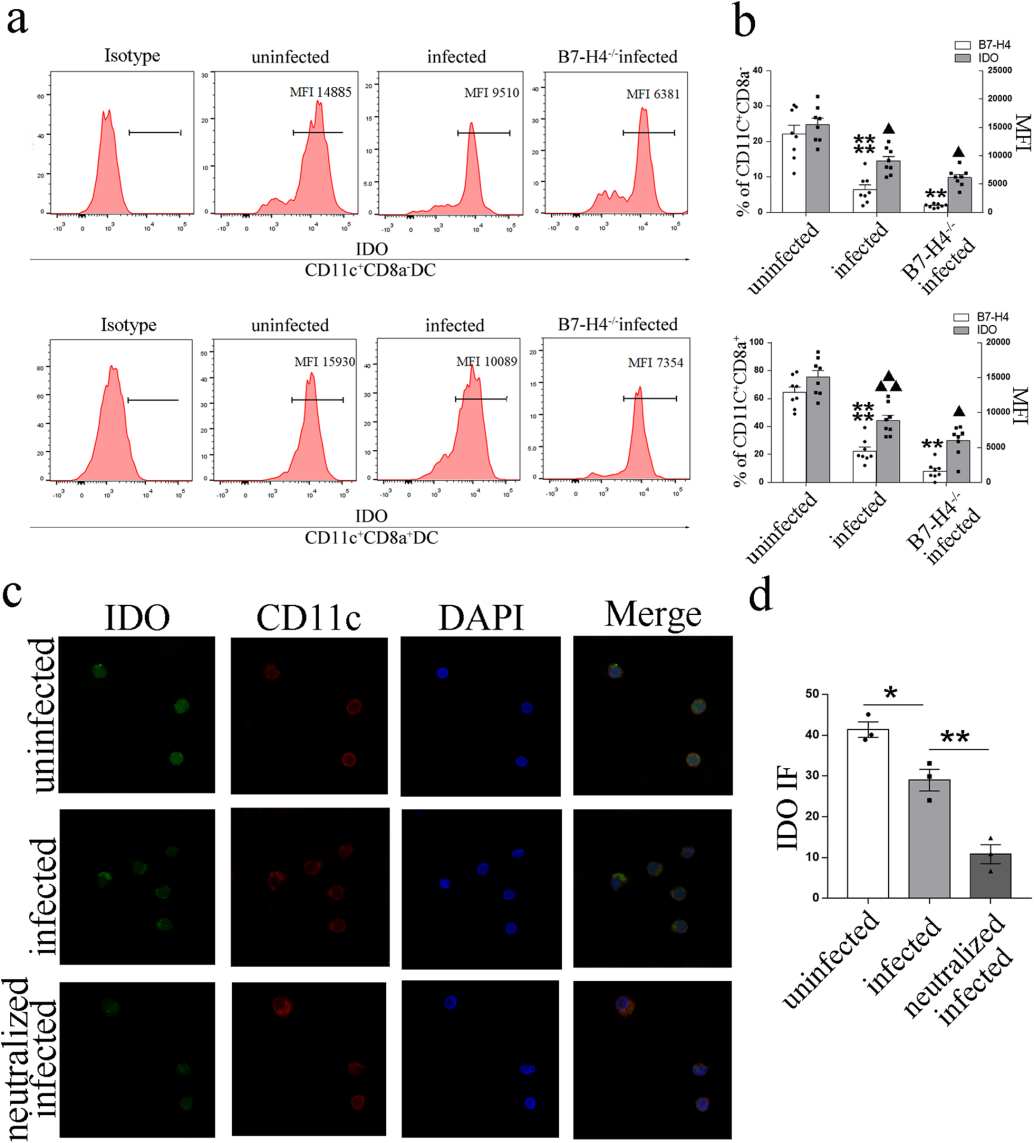
Reduction of B7-H4 on dDCs by *T. gondii* infection associated with the expression of the intracellular enzyme IDO. **a** Flow cytometry analyses of B7-H4 and IDO levels in CD11c^+^CD8a^−^ and CD11c^+^CD8a^+^ dDCs from WT uninfected, WT infected, and B7-H4^−/−^ infected groups of pregnant female mice. **b** Histogram analysis of B7-H4 and IDO expression changes in CD11c^+^CD8a^−^ and CD11c^+^CD8a^+^ dDCs from WT uninfected, WT infected, and B7-H4^−/−^ infected pregnant female mice. **c** Representative images of IF of IDO (green) expression levels in purified human dDCs from uninfected, infected, and B7-H4-neutralized infected pregnant female mice. DAPI (blue) labeling defined the morphology of nuclei. Scale bars, 10 μm. **d** Histogram analysis of IDO expression changes on purified human dDCs by immunofluorescence intensity among three groups. Data shown are means ± SD. At least six samples of human decidua in each group were assayed individually in flow cytometry and at least three samples of human decidua in each group were assayed individually in Western blot and IF by paired *t*-test for uninfected vs infected group, infected vs B7-H4 neutralized infected group; At least eight pregnant female mice in each group were assayed individually by unpaired *t*-test for uninfected vs infected group, infected vs B7-H4^−/−^ infected group

Then to explore the effect of *T. gondii*-induced reduction of B7-H4 on dDC function, the expression of IDO was analyzed. As shown in Fig. 5a, b, flow cytometry revealed that IDO was significantly decreased in both CD11c^+^CD8a^−^ (*t*-test: *t*_(14)_ = 2.952, *P* = 0.10105) and CD11c^+^CD8a^+^ (*t*-test: *t*_(14)_ = 5.145, *P* = 0.0001) dDC subsets in infected pregnant female mice compared with uninfected mic pregnant female mice, and it was further reduced (CD11c^+^CD8a^−^: *t*-test: *t*_(14)_ = 2.94, *P* = 0.0107, CD11c^+^CD8a^+^: *t*-test: *t*_(14)_ = 2.645, *P* = 0.0192) in B7-H4^−/−^ infected pregnant female mice compared with WT infected pregnant female mice. Similar results were shown in vitro, where IF (Fig. 5c, d) and Western blot analysis (Fig. 7a, c) revealed that IDO expression was reduced (IF: *t*-test: *t*_(4)_ = 3.779, *P* = 0.0194) in human dDCs after *T. gondii* infection, and it was further reduced (IF: *t*-test: *t*_(4)_ = 5.133, *P* = 0.0068) in the dDCs of the B7-H4-neutralized infected group.

### Reduction of B7-H4 by *T. gondii* infection affected IL-10 and IL-12 secretion in dDCs

**Fig. 6:**
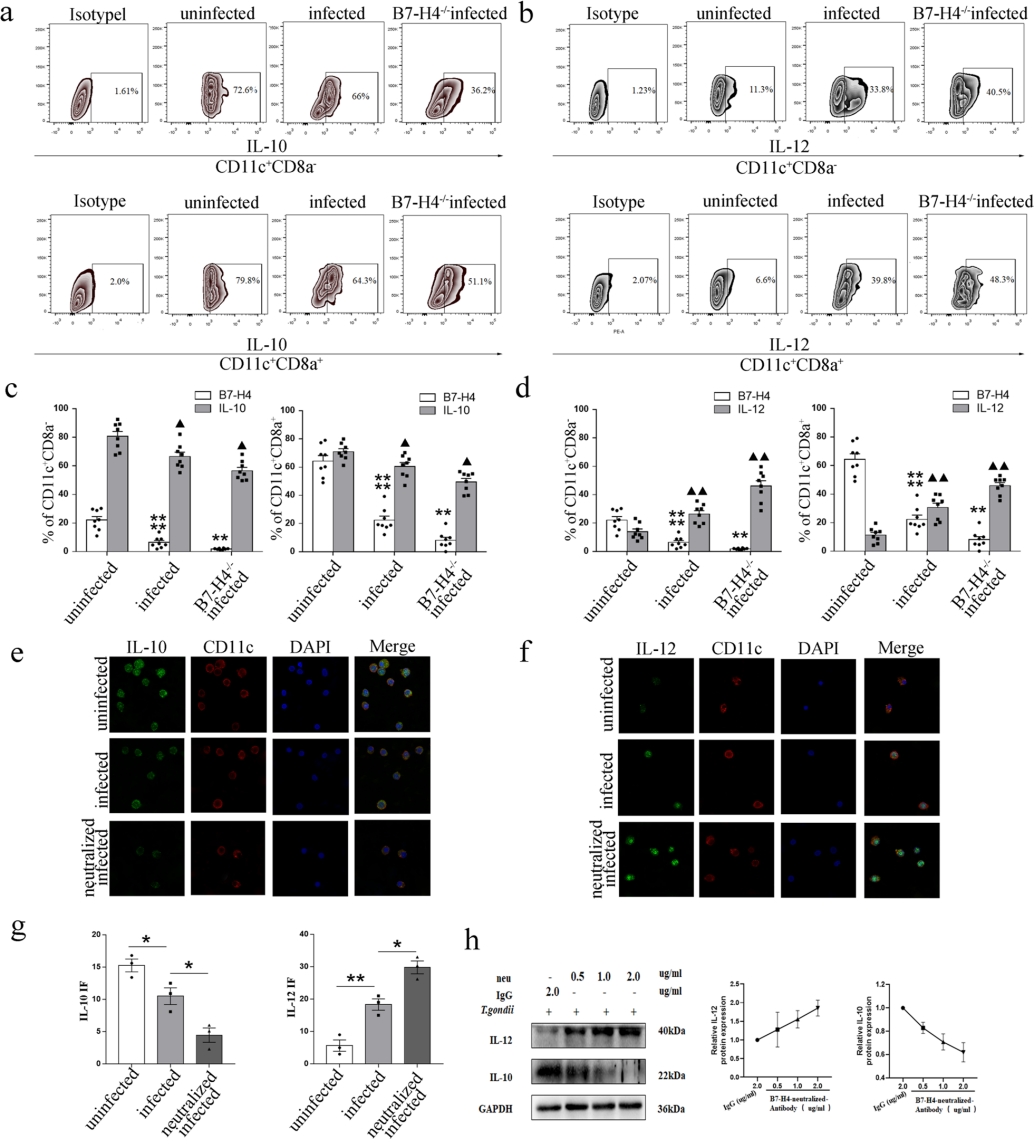
Reduction of B7-H4 on dDCs by *T. gondii* infection associated with the synthesis and secretion of IL-10 and IL-12. **a**, **b** Flow cytometry analyses of IL-10, IL-12, and B7-H4 levels in CD11c^+^CD8a^−^ and CD11c^+^CD8a^+^ dDCs from WT uninfected, WT infected, and B7-H4^−/−^ infected groups of pregnant female mice. **c**, **d** Histogram analysis of IL-10, IL-12, and B7-H4 expression changes in CD11c^+^CD8a^−^ and CD11c^+^CD8a^+^ dDCs from WT uninfected, WT infected, and B7-H4^−/−^ infected pregnant female mice. **e**, **f** Analysis of IL-10 and IL-12 (green) expression levels in uninfected, infected, and B7-H4-neutralized infected purified human dDCs by IF. DAPI (blue) labeling defined the morphology of nuclei. Scale bars, 10 μm. **g** Histogram analysis of IL-10 and IL-12 expression changes from purified human dDCs by immunofluorescence intensity. Data shown are means ± SD. At least six samples of human decidua in each group were assayed individually in flow cytometry and at least three samples of human decidua in each group were assayed individually in Western blot and IF by paired *t*-test for uninfected vs infected group infected vs B7-H4 neutralized infected group; and eight pregnant female mice in each group were assayed individually by unpaired *t*-test; uninfected and infected group, infected & B7-H4^−/−^ infected group. **h** Western blotting assay of IL-10 and IL-12 levels under *T. gondii* infection with varied concentrations (0, 0.5, 1.0, and 2.0 μg/ml) of B7-H4 neutralized antibody in purified human dDCs

Cytokines IL-12 and IL-10 were also the major functional molecules of dDCs. Hence, to address whether *T. gondii*-related reduction of B7-H4 expression affects cytokine secretion in dDC subsets, the expression levels of IL-12 and IL-10 were analyzed with IF and Western blot in vitro, and flow cytometry was used to analyze their expression in vivo. Flow cytometry results showed that the level of IL-12 expression in both CD11^+^CD8a^−^ (*t*-test: *t*_(14)_ = 4.029, *P* = 0.0012) and CD11c^+^CD8a^+^ (*t*-test: *t*_(14)_ = 4.127, *P* = 0.0010) dDC subsets increased robustly after *T. gondii* infection, and was further increased (CD11^+^CD8a^−^: *t*-test: *t*_(14)_ = 3.894, *P* = 0.0016, CD11^+^CD8a^+^: *t*-test: *t*_(14)_ = 3.739, *P* = 0.0022) in B7-H4^−/−^ pregnant mice infected with *T. gondii* compared with WT infected pregnant female mice (Fig. 6b, d). By contrast, the level of IL-10 expression was decreased in CD11^+^CD8a^−^ (*t*-test: *t*_(14)_ = 2.974, *P* = 0.0101) and CD11c^+^CD8a^+^ (*t*-test: *t*_(14)_ = 2.952,* P* = 0.105) DCs following *T. gondii* infection, and was further reduced (CD11^+^CD8a^−^: *t*-test: *t*_(14)_ = 2.586, *P* = 0.0215, CD11c^+^CD8a^+^: *t*-test: *t*_(14)_ = 2.935, *P* = 0.0109) in B7-H4^−/−^ pregnant mice infected with *T. gondii* compared with the WT infected group (Fig. 6a, c). Experiments in human dDC cultures showed similar results, with significantly increased expression of IL-12 (IF: *t*-test: *t*_(4)_ = 5.078, *P* = 0.0071) and significantly decreased IL-10 (IF: *t*-test: *t*_(4)_ = 2.9315, *P* = 0.0428) in dDCs after *T. gondii* infection; IL-12 (IF: *t*-test: *t*_(4)_ = 4.341, *P* = 0.0122) were further increased and IL-10 (IF: *t*-test: *t*_(4)_ = 3.546, *P* = 0.0239) were further decreased when infected group compared with neutralized infected group (Fig. 6e–g). Furthermore, infected human dDCs treated with anti-B7-H4 neutralizing antibody showed a higher level of IL-12 and a lower level of IL-10 than those of untreated infected dDCs in a dose-dependent manner (Fig. 6h).

### Reduction of B7-H4 on dDCs by *T. gondii* infection affected the expression of functional molecules IDO, IL-10, and IL-12 via the JAK2/STAT3 pathway

**Fig. 7:**
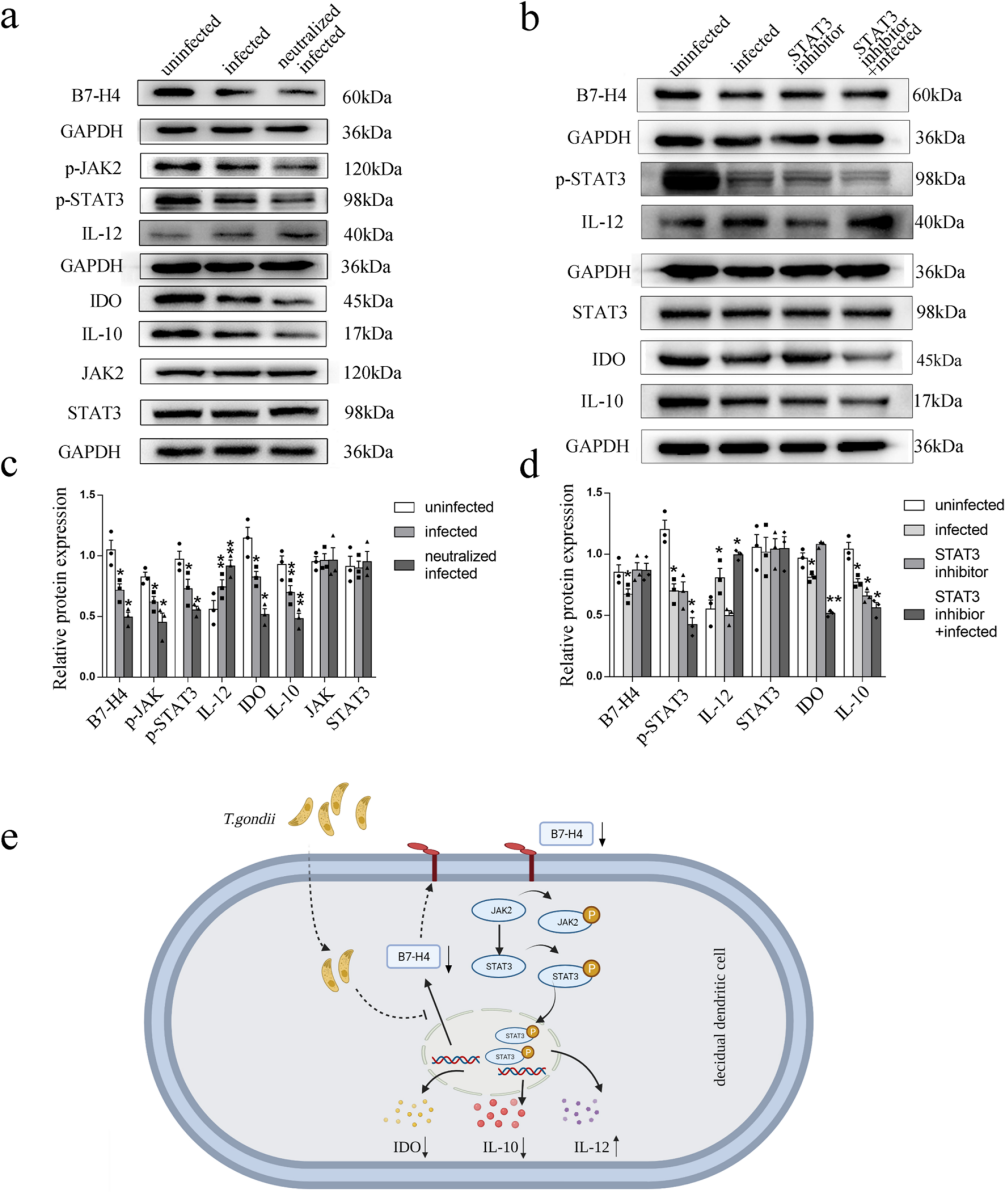
Reduction of B7-H4 on dDCs by *T. gondii* infection associated with the expression of IDO, IL-10, and IL-12 via regulation of the JAK2/STAT3 pathway. **a**, **c** Western blotting assay of B7-H4, IDO, IL-10, IL-12, JAK2, p-JAK2, STAT3, and p-STAT3 levels from uninfected, infected, and B7-H4-neutralized infected groups of purified human dDCs. **b**, **d** Western blotting assay analysis of B7-H4, IDO, IL-10, IL-12, STAT3, and p-STAT3 levels from uninfected, infected, STAT3 inhibitor-treated, or STAT3 inhibitor-treated infected groups of purified human dDCs. Data shown are means ± SD. At least three samples of human decidua in each group were assayed individually by paired *t*-test in Western blot; uninfected vs infected group, infected and B7-H4-neutralized infected group. **e** A pattern diagram of possible mechanisms by which *T. gondii* infection can lead to a decrease in B7-H4 on the dDC, ultimately leading to dysfunction of functional molecule

To further explore the detailed mechanism of the effect of B7-H4 signaling on IDO, IL-10, and IL-12 expression, the Janus kinase (JAK)-signal transducer and activator of transcription (STAT) signaling pathway was analyzed by STAT3 inhibition and Western blotting assay. Results showed the expression of phosphorylated JAK2 (p-JAK2) (*t*-test: *t*_(2)_ = 5.939, *P* = 0.0272) and phosphorylated STAT3 (p-STAT3) (*t*-test: *t*_(2)_ = 8.314, *P* = 0.0142) levels were both decreased in human dDCs after *T. gondii* infection, and further reduction (p-JAK2: *t*-test: *t*_(2)_ = 4.336, *P* = 0.0493, p-STAT3: *t*-test: *t*_(2)_ = 4.357, *P* = 0.0489) in B7-H4-neutralized infected group (Fig. 7a, c). To verify the effect of STAT3 mediating IDO and cytokines secretion, human dDCs were treated with a STAT3 inhibitor 2 h before *T. gondii* infection. The data showed that B7-H4 (*t*-test: *t*_(2)_ = 0.1443, *P* = 0 0.8985) expression was no different between the infected groups and STAT3-inhibitor infected group (Fig. 7b, d). Compared with the infected group, IDO (*t*-test: *t*_(2)_ = 17.68, *P* = 0.0032) and IL-10 (*t*-test: *t*_(2)_ = 4.768, *P* = 0.0413) expression in dDCs was further reduced and IL-12 (*t*-test: *t*_(2)_ = 6.571, *P* = 0.0224) expression was further increased in the STAT3-inhibitor infected group (Fig. 7b, d). Taken together, these results indicate that the reduction of B7-H4 expression in dDCs caused by *T. gondii* infection could affect the production of IDO and secretion of cytokines (IL-10 and IL-12) via the JAK2/STAT3 signaling pathway (Fig. 7e).

## Discussion

*Toxoplasma gondii* is an obligate intracellular protozoan that infects endotherms including humans, resulting in miscarriage, fetal death, or other abnormal outcomes during pregnancy [25]. Successful pregnancy is dependent on specific kinds of immune cells and their cytokines at the maternal–fetal interface that contribute to the implantation of the embryo, the decidualization of the endometrium, and maternal tolerance of the fetus [26]. Several studies have already demonstrated that *T. gondii* disrupts the immune microenvironment balance at the maternal–fetal interface and further leads to abnormal pregnancy [4–6]. Our previous studies reported that *T. gondii* infection causes abnormal expression of functional membrane molecules and abnormal production of cytokines by dDCs, macrophages, NK cells, or Tregs, and the dysfunction of these decidual immune cells contributes to the abnormal outcomes [4, 27, 28]. dDCs at the maternal–fetal interface mainly exert and maintain immune tolerance during a successful pregnancy [29]. Deficiency in dDC tolerance induces various pregnancy complications [30]. We recently reported that *T. gondii* infection reduces LILRB4 expression, thus impairing the tolerance function of dDCs and further resulting in abnormal pregnancy [24]. B7-H4, an immune inhibitory molecule, was initially thought to be involved in the negative regulation of T-cell-mediated immune responses by inhibiting proliferation and cytokine secretion [20]. It has also been reported that mDCs and pDCs express a high level of B7-H4 during normal pregnancy [23]. However, it remains unclear whether *T. gondii* infection reduces B7-H4 expression to cause dysfunction of dDCs and contribute to abnormal pregnancy.

In the present study, we showed that the expression level of B7-H4 on both CD11c^+^CD8a^−^ and CD11c^+^CD8a^+^ dDC subsets in pregnant female mice were significantly decreased after *T. gondii* infection. Similarly, B7-H4 expression was also significantly reduced on human dDCs after *T. gondii* infection. To explore whether B7-H4 expression participates in abnormal pregnancy outcomes caused by *T. gondii* infection, B7-H4^−/−^ infected pregnant female mice were established. The results of the present study revealed that the pregnancy outcomes of B7-H4^−/−^ infected pregnant female mice were worse than those of infected WT pregnant female mice, with more stillbirths and resorbed fetuses as well as smaller placental and fetal sizes. These results strongly suggest that B7-H4 expression is closely related to adverse pregnancy outcomes caused by *T. gondii* infection. However, whether the downregulation of B7-H4 expression caused by *T. gondii* infection could result in dDC dysfunction and the further details of its molecular mechanism still need to be clarified. To explore how reduction of B7-H4 expression induced by *T. gondii* infection affects the function of dDCs, the expression levels of functional molecules on dDCs including membrane molecules, intracellular enzymes, and cytokines were analyzed.

DCs are generally differentiated into distinct immunogenic or tolerogenic DC subsets. Previous studies have reported that the mouse myeloid DC (CD11c^+^CD8a^−^) subset mainly exerts tolerance to facilitate a successful pregnancy at the maternal–fetal interface, but the lymphoid DC (CD11c^+^CD8a^+^) subset exerts immune activation [14]. However, several studies reported that the three human dDC subsets, including PDC, MDC1, and MDC2, have an immature phenotype at the maternal–fetal interface that contributes to the maintenance of normal pregnancy [8]. CD80 and CD86 that are expressed on DCs provide co-stimulatory signals in T-cell activation [31], and they are quickly enhanced after encountering inflammatory or pathogen-derived mediators [32]. dDCs have an immature phenotype characterized by low expression of CD80, CD86, and MHC-II (HLA-DR) at the maternal–fetal interface to contribute to the maintenance of normal pregnancy [33, 34]. Our previous study demonstrated that the decrease in LILRB4 expression during *T. gondii* infection leads to upregulated expression of CD80, CD86, and MHC-II, further contributing to dDC dysfunction [24]. However, whether the reduction of B7-H4 expression after *T. gondii* infection could change the expression of membrane functional molecules and further lead to the dysfunction of dDCs was still unclear. For the in vivo study, B7-H4^−/−^ infected pregnant female mice were used. The data herein show that, in vivo, these functional membrane molecules (CD80, CD86, MHC-II) of dDCs in B7-H4^−/−^ infected pregnant female mice were increased compared with those of WT infected pregnant female mice. Similar results were observed in vitro, as treatment of the infected human dDCs with anti-B7-H4 neutralizing antibody upregulated the expression of functional membrane molecules compared with those of infected human dDCs without antibody treatment. Thus, the variation in the DC expression of functional membrane molecules may be due to the reduction of B7-H4 expression by *T. gondii* infection. Furthermore, *T. gondii* infection may impair the immune tolerogenic function of dDCs by reducing B7-H4 expression and may enhance immune-activated functions by increasing CD80, CD86, and MHC-II expression. Together these changes may further contribute to abnormal pregnancy outcomes.

Emerging evidence suggests that one of the specific immunosuppressive mechanisms of DCs occurs via the tryptophan metabolic enzyme IDO [35]. IDO participates in the catalytic decomposition of tryptophan and suppresses maternal T-lymphocyte activation to build maternal immune tolerance to the fetus [36]. The DCs of pregnant women express significantly higher levels of IDO than those of non-pregnant women in which IDO is nearly absent [10]. Some studies have demonstrated a link between the decreased expression of IDO at the maternal–fetal interface and various adverse pregnancy outcomes, including recurrent spontaneous abortion (RSA) and preeclampsia [37, 38]. In the present study, IDO synthesis in each subset of pregnant female mice dDCs was significantly decreased after *T. gondii* infection, and IDO expression was similarly decreased in human dDCs by *T. gondii* infection. Thus, these findings suggest that *T. gondii* infection may impair the tolerance function of dDCs via reduction of IDO expression. In B7-H4^−/−^ infected pregnant female mice, the IDO expression level was obviously decreased compared with that of infected WT pregnant female mice. Furthermore, IDO expression was reduced after blocking B7-H4 with a B7-H4-neutralizing antibody in infected human dDCs. These studies therefore clarified that the reduction of B7-H4 by *T. gondii* infection results in a decrease in IDO expression, which then impairs the tolerance function of dDCs, and this may be an important molecular mechanism of abnormal pregnancies. IL-10, a Th2 cytokine produced by many immune cells such as DCs, suppresses the activation of T cells, B cells, and NK cells to regulate the innate and adaptive immune responses [39]. Additionally, dDCs spontaneously release high levels of IL-10 at the maternal–fetal interface and play key roles in the induction of maternal immune tolerance to fetal antigens [40]. IL-12 is a Th1 cytokine and may induce a potent immune response by promoting the naïve T-cell shift to the Th1 phenotype [41]. At the maternal–fetal interface, dDCs secrete significantly higher levels of IL-10 than IL-12 [14]. This balance in IL-10 and IL-12 cytokine production is pivotal for building immune tolerance during a successful pregnancy, and disruption of this balance leads to abnormal pregnancy with fetal loss [8]. IL-12 secretion is elevated in dDCs after *T. gondii* infection, resulting in cytokine imbalance at the maternal–fetal interface and further contributing to abnormal pregnancy outcomes [5]. Our data show that *T. gondii* infection decreases the secretion of IL-10 and increases the secretion of IL-12 in dDCs. Moreover, we found significantly higher expression of IL-12 and lower IL-10 in B7-H4^−/−^ infected pregnant female mice and infected B7-H4-neutralized human dDCs than in those of infected dDC controls, demonstrating that the IL-10/IL-12 imbalance in dDCs may be due to the reduction of B7-H4 after *T. gondii* infection.

Research suggests that the JAK2/STAT3 pathway is involved in B7-H4-mediated IL-6 secretion in esophageal squamous cell carcinoma (ESCC) cells and enhances the growth and tumorigenicity of cells [42]. Accumulating evidence also suggests that STAT3 is involved in regulating the production of the intracellular enzyme IDO in DCs [43, 44]. Production of IL-10 is regulated by various transcription factors and signaling pathways, and STAT3 is an important regulator of IL-10 gene production by APCs as well as a negative regulator of DC function [45, 46]. Moreover, the presence of the transcription factor STAT3 inhibits the production of IL-12 in DCs [47]. To explore whether B7-H4 affects the DC expression of functional molecules via the JAK2/STAT3 signaling pathway during *T. gondii* infection, B7-H4-neutralizing antibody was used. In the present report, levels of activated p-JAK2 and p-STAT3 expression were lower in B7-H4-neutralized infected human dDCs than in those of the infected group as analyzed by Western blot, and changes in p-JAK2 and p-STAT3 expression were positively correlated with B7-H4 expression. Thus, our results indicate that B7-H4 may regulate the JAK2/STAT3 signaling pathway during *T. gondii* infection. To further explore whether the STAT3 signaling pathway is involved in the synthesis of IDO, IL-10, and IL-12 due to B7-H4 reduction by *T. gondii* infection, the human infected dDCs were treated with a STAT3 inhibitor. In the present study, we further show that treatment of human infected dDCs with a STAT3 inhibitor enhanced the production of IL-12 and reduced IL-10 and IDO expression. Additionally, B7-H4 expression showed no significant difference after inhibition of STAT3. This indicates that B7-H4 reduction caused by *T. gondii* infection regulates IDO synthesis and the production of IL-10 and IL-12 in dDCs via the JAK2/STAT3 signaling pathway, further leading to dDC dysfunction and contributing to abnormal pregnancy outcomes.

Studies have shown that *T. gondii* infection upregulated p-STAT3(705) expression in bone marrow-derived macrophages [48] and the A549 cell line [49]. For p-STAT3(727), however, no significant differences were found after RH *T. gondii* infection in human HaCaT cells [50]. To date, no studies about p-STAT3(727) expression have been reported in human dDCs with *T. gondii* infection. Our results showed that *T. gondii* infection led to reduced expression of p-STAT3(727) in dDCs. As we know, the maternal–fetal interface is a special tolerant and modulatory microenvironment, in which significant differences exist from the peripheral environment. Thus, the different cell types and special microenvironment might cause the differences in p-STAT3(727) expression during *T. gondii* infection.

## Conclusions

In summary, the results of the present study show that reduction of B7-H4 expression by *T. gondii* infection is correlated with adverse pregnancy outcomes as a result of dDC dysfunction, which is characterized by decreased secretion of the anti-inflammatory cytokine IL-10 and intracellular enzyme IDO and increased secretion of the pro-inflammatory cytokine IL-12. Moreover, the reduction of B7-H4 expression by *T. gondii* infection regulates IDO production and cytokine secretion of dDCs via the JAK2/STAT3 signaling pathway, further contributing to abnormal pregnancy outcomes.

## Supplementary Information

**Additional file 1: Figure S1.** Gating strategy for dDCs by flow cytometry. (**a**) In humans, the P1 gate is based on forward and side scatter (FSC -A and SSC-A) to remove dead cells and cell fragments. Then, myeloid DC type 1 P2 (MDC1, BDCA1^+^CD19^−^CD14^−^) were gated out using markers BDCA1, CD19 and CD14, myeloid DC type 2 P4 (MDC2, BDCA3^+^CD14^−^) were gated out using markers BDCA3 and CD14, and PDC P3 (BDCA2^+^CD123^+^) were gated out using markers BDCA2 and CD123. (b) In pregnant female mice, subset P1 were gated out using FSC-A and SSC-A. And then, CD11c^+^CD8a^+^ DC subset P4 and CD11c^+^CD8a^+^DC subset P3 were further subdivided using markers CD11c and CD8a.

**Additional file 2: Table S1.** Antibody catalog no., vendor, clone, fluorochrome, concentration used in this study.

## Data Availability

All data generated in this study are presented within this published article.

## Abbreviations

dDCs: Decidual dendritic cells
DCs: Dendritic cells
IDO: Indoleamine 2,3-dioxygenase
NK cells: Natural killer cells
Tregs: Regulatory T cells
pDCs: Plasmacytoid DCs
MDC1: Myeloid DC type 1
MDC2: Myeloid DC type 2
APCs: Antigen-presenting cells
WT: Wild-type
SPF: Specific-pathogen-free
Gd: Gestational day
MEM: Minimum essential medium
i.p.: Intraperitoneally
PBS: Sterile phosphate-buffered saline
SEM: Scanning electron microscope
IF: Immunofluorescence
DAPI: 4′,6-Diamidino-2-phenylindole
PMSF: Phenylmethylsulfonyl fluoride
ECL: Chemiluminescence
JAK2: Janus kinase 2
STAT: Signal transducer and activator of transcription
p-JAK2: Phosphorylated JAK2
p-STAT3: Phosphorylated STAT3
RSA: Recurrent spontaneous abortion
ESCC: Esophageal squamous cell carcinoma
