# Supplementary figures and images for: B7-H4 reduction induced by Toxoplasma gondii infection results in dysfunction of decidual dendritic cells by regulating the JAK2/STAT3 pathway
Source: Parasit Vectors. 2022 May 3;15:157. doi: 10.1186/s13071-022-05263-1 (PMC9066748; doi:10.1186/s13071-022-05263-1)

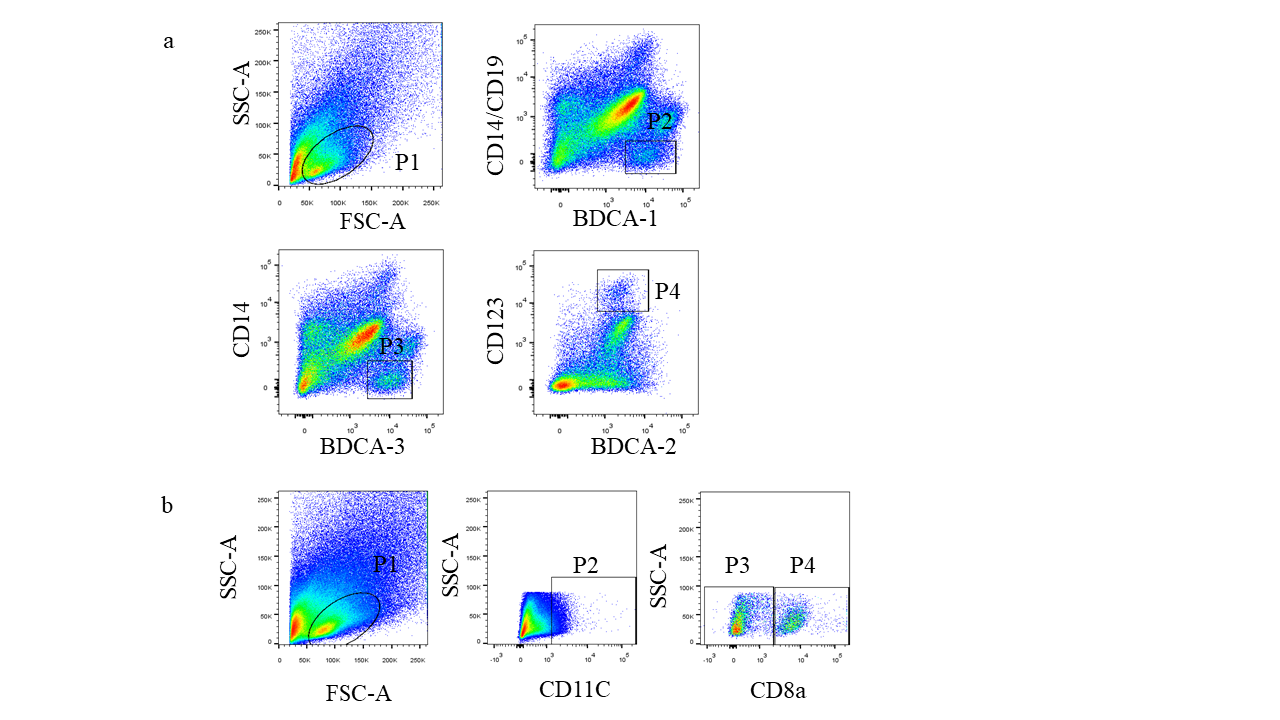

Supplement: Supplementary file 1 — Additional file 1: Figure S1. Gating strategy for dDCs by flow cytometry. (a) In humans, the P1 gate is based on forward and side scatter (FSC -A and SSC-A) to remove dead cells and cell fragments. Then, myeloid DC type 1 P2 (MDC1, BDCA1+CD19−CD14−) were gated out using markers BDCA1, CD19 and CD14, myeloid DC type 2 P4 (MDC2, BDCA3+CD14−) were gated out using markers BDCA3 and CD14, and PDC P3 (BDCA2+CD123+) were gated out using markers BDCA2 and CD123. (b) In pregnant female mice, subset P1 were gated out using FSC-A and SSC-A. And then, CD11c+CD8a+ DC subset P4 and CD11c+CD8a+DC subset P3 were further subdivided using markers CD11c and CD8a. [file 13071_2022_5263_MOESM1_ESM.tif]
